# Fluorescent Materials for Monitoring Mitochondrial Biology

**DOI:** 10.3390/ma14154180

**Published:** 2021-07-27

**Authors:** Yeonjeong Chu, Jisoo Park, Eunha Kim, Sanghee Lee

**Affiliations:** 1 Creative Research Center for Brain Science, Brain Science Institute, Korea Institute of Science and Technology, Seoul 02792, Korea; duswjd1415@ajou.ac.kr (Y.C.); chunjey1110@gmail.com (J.P.); 2 Department of Molecular Science and Technology, Ajou University, Suwon 16499, Korea; 3 Department of HY-KIST Bio-Convergence, Hanyang University, Seoul 02792, Korea

**Keywords:** mitochondria, fluorescence imaging, fluorescent chemical probes, fluorescent nanosensors, mitochondria-targeting peptides

## Abstract

Mitochondria play important roles in diverse cellular processes such as energy production, cellular metabolism, and apoptosis to promote cell death. To investigate mitochondria-associated biological processes such as structure, dynamics, morphological change, metabolism, and mitophagy, there exists a continuous demand for visualizing and monitoring techniques elucidating mitochondrial biology and disease-relevancy. Due to the advantages of high sensitivity and practicality, fluorescence phenomena have been most widely used as scientific techniques for the visualization of biological phenomena and systems. In this review, we briefly overview the different types of fluorescent materials such as chemical probes, peptide- or protein-based probes, and nanomaterials for monitoring mitochondrial biology.

## 1. Introduction

Mitochondria play pivotal roles in supplying cellular energy in the form of adenosine triphosphate (ATP) via oxidative phosphorylation [1]. Recent enthusiastic efforts have revealed that this ‘powerhouse of the cell’ is indeed the hub of intracellular signaling, energetics, and redox balance [2,3,4]. Considering the important role of mitochondria in calcium homeostasis, fatty acid synthesis, and biogenesis of the heme and iron-sulfur proteins, the interconnection between mitochondria and cellular signaling is essential [5,6]. Mitochondria provide energy in the form of ATP for cell survival; however, mitochondria are also actively implicated in apoptosis to promote cell death [7]. Since mitochondria are key regulators of apoptosis, they participate in developmental processes and aging [8]. Therefore, mitochondrial dysfunction is associated with aging-related phenomena including metabolic disorders, cardiomyopathies, and neurodegeneration [9]. Consequently, there is a high demand for understanding mitochondrial biology as a new frontier in health and disease.

Mitochondria are double-membraned organelles. The mitochondrial inner membrane has characteristic folds, called cristae, providing a large amount of surface area for chemical reactions, and it is enclosed by a permeable mitochondrial outer membrane, which completes the double membrane-bound architecture of mitochondria [10]. Mitochondria undergo dynamic movement inside cells by fusion and fission and build large interconnected intracellular networks, a process which is called mitochondrial dynamics [11]. It is generally believed that mitochondrial dynamics allows the cell to respond to cellular environmental changes, and results in the cell-type-specific appearance of the mitochondrial morphology. In addition, recent studies have demonstrated that mitochondrial dynamics is important for understanding multiple biological processes, and dysfunctions of mitochondrial dynamics could trigger several human diseases [8,12]. Therefore, monitoring mitochondrial morphology could provide a clue to learn many different biological processes for human diseases.

Mirroring these important roles of mitochondria in biological systems, there exists considerable interest in the development of new material for monitoring mitochondrial structure and function [4,12]. With a high sensitivity and signal-to-noise ratio, simple operation, practicality, good selectivity, and the capability for real-time detection, fluorescence has been utilized as a promising technique. From monochromatic fluorescent probes to ratiometric, multi-photon probes, and target-switchable fluorescent probes, a variety of fluorescent chemical probes has been developed [13]. Due to the high extinction coefficients, quantum yields, and modularity, the engineering of multicolored fluorescent proteins has enabled the development of various protein sensors [14]. Based on the high loading capacity and availability in multimodal imaging or sensing, there exists a continually growing attention for fluorescent nanomaterials in bioimaging [15]. The application of these fluorescent materials in mitochondrial research has disclosed a new area for monitoring mitochondrial biology in vitro and in vivo [4,16,17,18,19]. For these purposes, versatile fluorescent materials such as chemical compounds, peptide-conjugated fluorophores, engineered fluorescent proteins, and fluorescent nanomaterials have been developed and applied in monitoring mitochondrial structure and function. In this review, we briefly overview the various types of fluorescent materials for monitoring mitochondria and their application in biological phenomena.

## 2. Fluorescent Chemical-Based Mitochondria Probes

**Figure 1:**
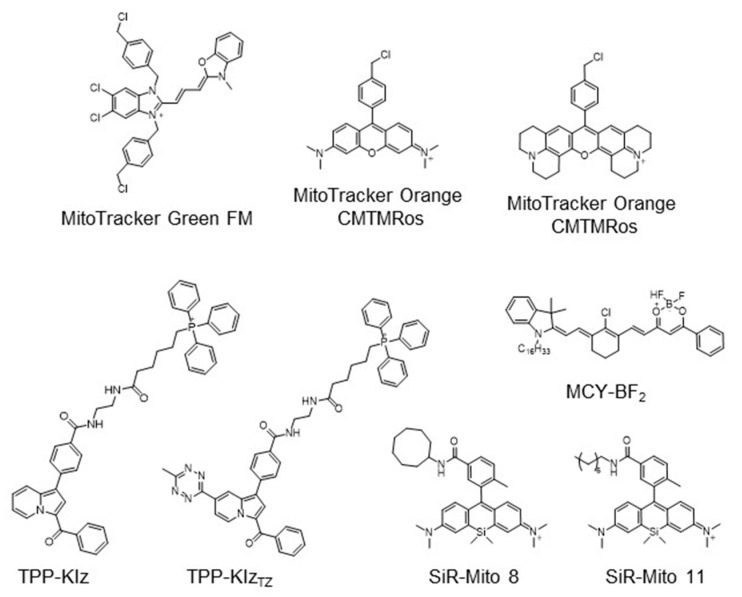
Chemical structures of various fluorescent chemical probes for mitochondrial biology [25,26,29,30,31,32,33].

The strategy for the development of fluorescent chemical probes for monitoring mitochondria-associated biological events is usually presented by conjugation of the mitochondria-targeting motif with an organic fluorophore using a suitable linker (Figure 1). As a part of anchoring mitochondria, the lipophilic cation is mainly considered as a key motif for the accumulation of the chemical in the mitochondrial matrix [20]. A delocalized cationic character in conjugated systems such as aromatic rings is essential for the accumulation of chemicals in mitochondria [20]. Therefore, the manipulation of the lipophilic character of chemical probes can affect their ability for targeting efficiency toward mitochondria. Based on structural insights, various kinds of fluorescent probes bearing lipophilic cations in diverse fluorophores such as boron-dipyrromethene (BODIPY), Rhodamine, cyanine-based Alexa-fluor, and acedan have been developed and are already commercially available to monitor mitochondria or membrane potential, e.g., MitoTracker Green, MitoTracker Orange, MitoTracker Red, and MitoTracker Deep Red (Figure 1) [19,21,22,23,24,25,26,27]. In particular, the last three dyes—MitoTracker Orange, Mitotracker Red, and MitoTracker Deep Red—are available in super-resolution imaging like stochastic optical reconstruction microscopy (STORM) [19,28].

The application of fluorogenic organic dyes that induce ‘turn-on’ fluorescence by a specific event in the case of localization in mitochondria has reduced background fluorescence and obtained a high signal-to-noise ratio [23,29]. Herein, we introduce interesting examples for fluorogenic mitochondria chemical probes and systematic studies for the regulation of hydrophobicity targeting mitochondria. In addition, we focus on the development of versatile fluorescent chemical probes for monitoring mitochondrial metabolites and membrane potential.

### 2.1. Triphenylphosphonium (TPP) Group Embedded Fluorogenic Probes

As a mitochondria-targeting motif, TPP is the most widely used functional group in the application of chemical probes as well as other fluorescence materials [4,20,34,35]. Due to the critical impact of the fluorogenic approach, we briefly introduce recent studies in the development of chemical probes using the fluorogenic dye-bearing TPP group. Fluorogenic properties of the desired probe only induce the enhancement of fluorescent intensity when the probes are targeting mitochondria; therefore, it is considered as a key feature of fluorescent technique to enhance resolution and practicality.

**Figure 2:**
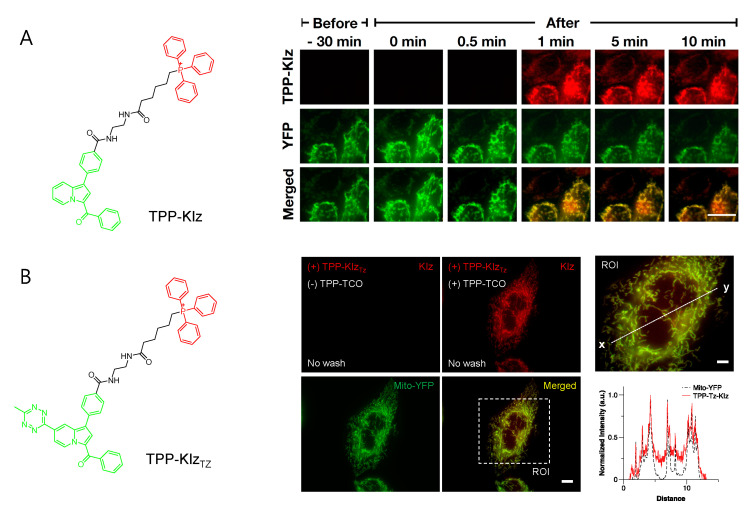
Washing-free mitochondrial imaging by fluorogenic indolizine mitochondria probes. (**A**) Structure of TPP-KIz and time-lapse imaging of TPP-KIz (1 nM) in Chang liver cells. Adapted with permission from ref. [29]. Copyright 2020 American Chemical Society. (**B**) Structure of TPP-KIz_Tz_ and fluorogenic imaging of TPP-KIz_Tz_ (100 nM) after addition of TPP-TCO (100 nM) in Chang liver cells. Green and red in structure indicate the fluorophore and mitochondrial-targeting moiety, respectively. Overexpression of Mito-YFP was compared to evaluate mitochondrial targeting efficiency in both figures. Adapted with permission from ref. [30]. Copyright 2021 Elsevieer B. V.

Conjugation with aggregation-induced emission luminogens (AIEgen) with TPP allowed a highly efficient fluorogenic mitochondria-targeting probe (Figure 2A) [29]. Kaleidolizine (KIz) as a molecular platform for AIEgen phenomena was reported to increase fluorescent intensity by over 120-fold when it aggregated [29]. As a versatile tool for monitoring biological systems, TPP-KIz was designed for bioimaging mitochondria and successfully visualized mitochondria in live cells within a minute without any additional washing step [29]. The further application of bioorthogonal chemistry for AIEgen allowed spatiotemporally controlled-mitochondria visualization with minimal perturbation of the cellular environment. Using the advantage of fast kinetics and selectivity of the reaction, the study of inverse electron-demand Diels–Alder (iEDDA) reactions between *trans*-cyclooctene (TCO) and tetrazine (Tz) combined with AIEgen-based KIz fluorophores and mitochondrial targeting TPP groups was reported (Figure 2B) [30]. Without TPP-TCO, TPP-KIz_Tz_ was quenched in a basal state, but iEDDA reactions with TPP-TCO dramatically increased the fluorescence of the product [30]. Bioorthogonal reactions between TPP-KIz_Tz_ and TPP-TCO suggested extremely specific and hypersensitive washing-free mitochondrial imaging by two-step mitochondrial targeting and the fluorescent turn-on process [30].

### 2.2. Hydrophobicity-Driven Accumulation in Mitochondria for Chemical Probes

Hydrophobic regulation of compounds is critical to access mitochondria. Silicon-rhodamine (SiR) dye has been considered a useful fluorophore due to its cationic character [31]. Sung et al. investigated the systematic effect of hydrophobicity with a silicon-rhodamine fluorescent core skeleton in view of mitochondrial targeting efficiency [31]. By modification of hydrophobic functional groups ranging from 2.29 to 6.33 of cLogP, mitochondria-targeting efficiency was highly correlated with increasing hydrophobicity and optimal cLogP in silicon-rhodamine, which was analyzed from 5.50 to 6.33 [31]. Additionally, SiR-Mito 8 showing enhanced mitochondrial localization was used to monitor mitochondrial membrane potential in Hep3B liver cancer cells [31]. Based on this study, a SiR-Mito 11 bearing *n*-octyl functional group was further developed with increasing selectivity on brain tumor cells, which further demonstrated a theragnostic effect on glioma cells of SiR-based mitochondrial fluorescent probes [32]. The polarity-sensitive Near-Infrared (NIR) fluorescent dye, MCY-BF2, bearing an *n*-hexadecyl group as a lipophilic cation group for mitochondria targeting was reported [33]. MCY-BF2 accumulated in mitochondria interacting with a diphosphatidylglycerol lipid in the inner membrane of the mitochondria and dramatically increased NIR fluorescence in response to mitochondrial polarity based on the change in microenvironment dielectric constant [33].

### 2.3. Targeting Metabolites in Mitochondria

Mitochondria are major organelles that regulate various cellular metabolism processes related to the redox system for regulation of antioxidants, which indicates that numerous metabolites exist and affect the balance of mitochondria. Therefore, various sensors of mitochondrial metabolites have been developed (Table 1).

Mitochondria are considered a sink of cellular H_2_O_2_ and play a major role in antioxidant defense systems. Mitochondria peroxy yellow 1, MitoPY1, was developed to visualize mitochondrial H_2_O_2_ in live cells [36,37]. MitoPY1 was designed through a bifunctional fluorescent molecule with boronate as a peroxide-responsive group and TPP as a mitochondrial-targeting moiety [36]. The boronate of MitoPY1 was deprotected in response to mitochondrial H_2_O_2_, thereby resulting in the oxidized probe, MitoPY1ox, which increased fluorescent intensity and allowed the efficient visualization of localized changes in H_2_O_2_ in mitochondria [36,37].

Glutathione is the major resource of thiols in biological systems, playing an antioxidant role in mitochondria as well as the entire organelle. The level of glutathione is highly associated with cellular dysfunction and disease status. Two-photon probe MT-1 for detecting mitochondrial thiols was reported based on the fluorescence resonance energy transfer (FRET) system [38]. MT-1 consisted of biocompatible naphthalimide fluorophores as FREP donors, cationic rhodamine B fluorophores as FRET accepters/mitochondrial targeting groups, and a 2,4-dinitrobenzenesulfonyl group for thiol detection, thereby effectively detecting the mitochondrial thiols represented by glutathione in live cells and tissue systems [38]. In particular, the photophysical properties of naphthalimide used by MT-1 exhibited the two-photon excitation around 800 nm and MT-1 was available in the two-photon imaging technique [38].

Malondialdehyde is one of the products of α,β-unsaturated reactive carbonyl species and is considered an oxidative stress marker. Mitochondria are major organelles for regulating oxidative stress and produce a high level of malondialdehyde. Mito-FMP was reported as a chemical probe for monitoring mitochondrial MDA that conjugated with a hydrazine moiety as a malondialdehyde recognition site and TPP as a mitochondrial targeting moiety in benzoxadiazole fluorophores [39]. Mito-FMP was a ‘turn-on’ fluorescent probe triggered by the production of a cyclized pyrazole in the presence of malondialdehyde and enabled the visualization of the cellular malondialdehyde in live cells [39].

Although nitroxyl (HNO) is a signaling agent for nitric oxide and is highly related to mitochondrial nitric oxide metabolism, direct detection of HNO is challenging due to its labile property. For detecting mitochondrial HNO in physiological conditions, a NIR chemical probe, MitoHNO, was developed [40]. MitoHNO including a 2-(diphenylphosphino)benzoyl group as the HNO recognition moiety and a merocyanine fluorophore with a lipophilic indolium as a mitochondria-targeting fluorophore precursor [40]. The reaction between MitoHNO and HNO induced the aza-ylide intermediate, then subsequently formed NIR fluorophores by Staudinger ligation that successfully confirmed the detection of HNO in mitochondria in live cells [40].

### 2.4. Targeting Mitochondrial Membrane Potential (MMP)

During the process of energy production in mitochondria, the concentration of various ions including protons is distributed on inner and outer membranes of mitochondria, which results in mitochondrial membrane potential [13,41]. The integrity of MMP is highly related to mitochondrial functions and there is a lot of evidence that the abnormality of MMP is associated with human diseases such as Parkinson’s disease, Alzheimer’s disease, and cancer [7,9,11,13,42,43]. Therefore, fluorescent probes for detecting MMP provide a great research tool for mitochondrial biology.

Commercially available MitoTracker Orange and JC-1 are widely used to detect MMP by an accumulation in membrane potential and are used to visualize mitochondria in the monochromatic and the ratiometric modes, respectively (Figure 1) [25,44]. In addition, an AIEgen strategy was applied to develop a fluorescent chemical probe for monitoring the membrane potential difference. Zhao et al. reported an AIE probe TPE-Ph-In for monitoring MMP with a tetraphenylethylene (TPE) as an AIE unit and the incorporation of indolium for targeting mitochondria [45]. TPE-Ph-In enabled the measurement of the level of MMP from the basal state to an increase or decrease. By oligomycine treatment, the increased MMP led to accumulation of TPE-Ph-In in mitochondria and induced aggregation and fluorescence, whereas the decreased MMP in response to carbonylcyanide 3-chlorophenylhydrazone treatment reduced the fluorescence of TPE-Ph-In by releasing from mitochondria to cytosole [45]. With the advantages of enhanced photostability and avoiding the quenching effect, the red-emitting probe, TPE-Ph-In, suggested the potential ability for a mitochondria probe [13,45].

## 3. Peptide- or Protein-Based Mitochondria Probes

Biomaterials such as peptides or proteins are major resources for the development of imaging tools for mitochondria. We focused on the recent approach for peptide- or protein-based strategies for mitochondrial visualization and their therapeutic application.

### 3.1. Application of Mitochondria-Targeting Peptide

**Figure 3:**
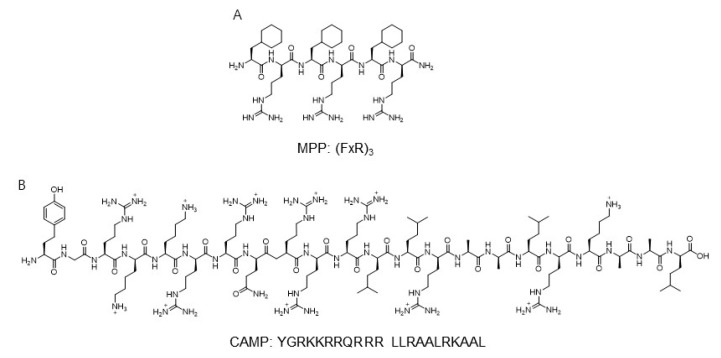
The sequence of mitochondria-targeting peptide MPP and CAMP [46,47]. Structures from references [46,47]. (**A**) The sequnce of MPP; (**B**) The sequnce of CAMP.

Based on the modularity and synthetic utility, peptide-based mitochondrial targeting motifs including both hydrophobic and cationic amino acids were used for application for the development of mitochondria probes. The mitochondria-penetrating peptide (MPP) based on a six-residue combination such as cationic arginine and artificial amino acid, cyclohexylalanine, was reported as a mitochondria carrier targeting the mitochondrial matrix (Figure 3) [46].

A systematic study involving modification of hydrophobicity using an artificial amino acid elucidated that the charge-driven cellular uptake across the plasma and mitochondrial membrane was important in the localization for mitochondria. The sequence of Fx-r-Fx-K-Fx-r-Fx-K and Fx-r-Fx-K (Fx and r indicate cyclohexylalanine and d-arginine, respectively)-linked thiazole orange dye showed a high Pearson’s correlation coefficient, which suggested the localization of peptide derivatives in mitochondria [46]. Additionally, the fluorescently labeled mt-Cbl was developed to lead the specific delivery of the DNA alkylating agent, nitrogenmustard chlorambucil (Cbl) [48]. The MPP peptide, (FxR)3, was conjugated with chlorambucil to C-terminal Lys residue and thiazole orange as a fluorophore to the N-terminal [48]. By mitochondria-directed activity, mt-Cbl improved the potency against various leukemic cells and confirmed apoptotic activity in drug-resistant cells [48].

Another mitochondria-targeting peptide sequence was developed to deliver protein to mitochondria (Figure 3). By in silico analysis, a novel cell-penetrating artificial mitochondria-targeting peptide (CAMP) was designed based on the structural insight between human immunodeficiency virus (HIV-1) trans-activator of transcription (TAT) peptide and the mitochondria-targeting sequence [47]. The sequence of CAMP is YGRKKRRQRRR LLRAALRK_AAL (the underscore indicates the cleavage site predicted by MitoProtII) [47]. The fusion of CAMP with EGFP or hMT1A allowed the delivery of these cargo proteins to mitochondria, then CAMP was efficiently cleaved by mitochondrial metalloprotease and released cargo proteins in cellular and in vivo mouse models [47]. These results demonstrate the capability of mitochondria-specific delivery systems using mitochondrial targeting peptides.

### 3.2. Protein for Visualization of Mitochondrial Biology

Historically, the engineered fluorescent protein has enabled real-time visualization for the protein of interest with high resolution and sensitivity. The diverse pH range of the mitochondria matrix and pH alteration induced by various perturbations such as breaking of calcium homeostasis or mitophagy have been considered as key events to study mitochondrial biology.

The mitochondrial alkaline pH indicator, mtAlpHi, which is an engineered green fluorescent protein with around 8.5 of pKa, selectively targeted mitochondria and changed fluorescence intensity in response to a pH range of 7–11 [49]. Based on the advantages of reversible response and lack of toxicity, mtAlpHi allowed monitoring the dynamics of mitochondrial pH by calcium uptake [49].

Mitophagy is a type of autophagy process that specifically removes the dysfunctional mitochondria by lysosomal degradation [50]. mt-Keima is the widely used fluorescent imaging technique to monitor mitophagy in in vitro and in vivo systems [17]. The characteristic features of Keima, such as ratiometric pH-sensitive fluorescence, allow the detection of protein location in the mitochondria (pH ~ 8.0) or the lysosome (pH ~ 4.5) by differentiated fluorescence for multicolor imaging [51]. Further application of mt-Keima discriminated whether the mitochondria were in the cytoplasm or in acidic lysosome, which allowed the monitoring of mitophagy in cells [52]. Moreover, the development of an mt-Keima transgenic mouse enabled the measurement of mitophagy in an in vivo system [53].

Due to the advantages of fast kinetics and high efficiency, bioorthogonal chemistry is widely used to label biomolecules mainly using click-chemistry without any interference in native biological processes [54]. Copper-free click chemistry such as iEDDA reactions using Tz and TCO disclosed the various applications to visualize biomolecules with fluorophore-linked Tz or TCO [55]. The incorporation of unnatural amino acids into the target protein, followed by fluorophore labeling using bioorthogonal reactions, suggested a feasible solution for monitoring mitochondrial protein [56]. By reprogramming the genetic codon, mitochondrial protein MITRAC12, which is an integral inner membrane protein, was modified by incorporation of TCO-L-lysine and subjected to iEDDA cycloaddition with a Star580-tetrazine as a fluorescent tag [57]. With the advantage of TCO-Tz bioorthogonal reactions like fast reaction kinetics, efficient reaction yield, and feasibility in physiological conditions, this approach allowed visualization of mitochondrial proteins in super-resolution imaging with a single cell level.

## 4. Fluorescent Nanomaterials for Mitochondria

**Figure 4:**
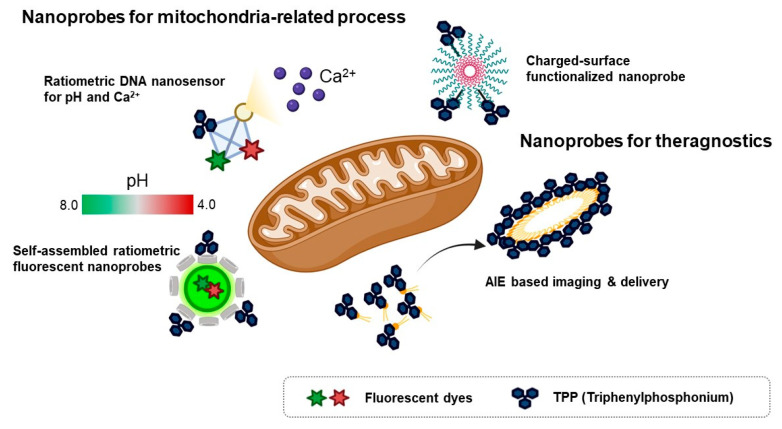
Schematic diagram for various types of mitochondria-targeting fluorescent nanoprobes [60,61,62,63]. Figure was depicted from the concept of individual fluorescent material in references [60,61,62,63].

Because of the loading capacity for various materials like drugs, fluorescent dyes, responsive units for an analyte, or mitochondria-targeting motifs at the same time, nanomaterials are considered valuable tools for theragnostic or dual-sensor systems for multiple analytes in mitochondria. Considering the regulation of complex biological processes like bioenergetic functions and redox homeostasis, the development of fluorescent tools for monitoring multiple functions that occur in mitochondria has been required in the field of mitochondria [7]. In addition, mitochondria contribute to cancer growth and survival, thereby they are regarded as a potential therapeutic target for various cancers [7,58]. Moreover, mitochondrial oxidative stress is a major pathological issue in neurodegenerative diseases such as Alzheimer’s disease [59]. Therefore, the fluorescent material for both monitoring and regulating mitochondrial function in parallel is valuable to study mitochondria-related disease. In this context, various types of nanomaterials have been developed for monitoring multiple functions of mitochondria and delivery agents to cure cancer (Figure 4).

To investigate multiple physiological events in mitochondria, a ratiometric DNA nanosensor for simultaneous monitoring of both calcium and pH in mitochondria was developed [60]. A tetrahedron DNA-based nanoprobe was conjugated with four components including NIR emissive carbon dots bearing a calcium ligand (CD@Cal), pH-responsive fluorescein, TPP as a mitochondria-targeting motif, and an AF660 as a reference fluorophore for quantitative analysis [60]. Without any interference, a tetrahedron DNA nanoprobe measured calcium and pH simultaneously in vitro and quantified mitochondrial calcium concentration and pH in the neuron by real-time imaging [60]. However, self-assembled ratiometric fluorescent nanoprobes (SRFNPs) for monitoring mitochondrial pH were reported [61]. The preparation of the probe is based on a self-assembly mechanism by host-guest interaction between β-cyclodextrin polymer (β-CDP) as the host backbone and adamantine as the guest molecules [61]. Three different components, including fluorescein, rhodamine, and TPP, were introduced to the host backbone via conjugating with adamantane. Since fluorescein has pH-sensitive fluorescent intensity, the intensity ratio between fluorescein and rhodamine quantitatively reported the pH of the mitochondria in a ratiometric way. In this study, adamantane-conjugated TPP was used as a mitochondria targeting moiety [61]. SRFNP was successfully applied to monitor mitochondrial pH in the range pH 4.0–8.0 and low toxicity of SRFNP revealed the biocompatibility of the probes for live cells [61].

A theragnostic approach for tumor targeting, imaging, and drug delivery with mitochondria-targeting nanoparticles was reported [62]. In this study, the author employed the AIE approach to display playing multiple roles in selective drug delivery, anticancer activity, and mitochondria targeting, showing potential ability for theragnostic tools in cancer therapy [62]. A self-assembled nanoparticle with cyanostilbene and a long alkyl chain was designed for targeting mitochondria by incorporation of a TPP moiety and revealed the accumulation in mitochondria by AIE phenomena [62]. Moreover, encapsulation of doxorubicin was successfully delivered to mitochondria and induced anti-cancer activity in both cell-based and in vivo systems [62]. Furthermore, photothermal properties of hydrophilic nanoparticles are expected to be a theragnostic approach based on photothermal therapy and photothermal/photoacoustic imaging [64]. Wang et al., reported Mito-BDP5 nanoparticles based on a purely organic BODIPY core with a modification by TPP and an ethylene glycol chain which successfully visualized mitochondria in HeLa cells and tumors in mice by photothermal and photoacoustic imaging with good bioavailability and enhanced permeability and retention effects [64]. TPP-based mitochondria-targeting graphene oxide nanocomposites loaded with indocyanin green TPP-PPG@ICG suggested a new class of fluorescence imaging-guided phototherapy [65]. Due to the preferential accumulation of TPP-PPG@ICG in tumors and availability in NIR light sources, this nanocomposite proved a therapeutic potential with enhanced photothermal efficacy and suppressed ATP production, which led to overcoming drug resistance [65]. Besides cancer therapy, mitochondria-targeting nanoparticles can be used as theragnostic tools for neurodegenerative disease. Ceria nanoparticles are known for their antioxidant activity by scavenging reactive oxygen species [59]. Mitochondria-targeting TPP-conjugated ceria nanoparticles were localized to mitochondria and suppressed neuronal death by regulating reactive oxygen species of damaged mitochondria in an Alzheimer’s disease mouse model [59].

To improve photostability and reduce photobleaching, CdSe/ZnS-based quantum dots and iron-oxide-based nanoprobes, which were coated with polyacrylate and covalently linked with TPP, were reported as functionalized nanoprobes for imaging mitochondria [63]. It was confirmed that inorganic nanoparticle quantum dot-TPPs with low surface charge and high colloidal stability enhanced mitochondrial targeting efficiency with low nonspecific binding and bypass endosomal trafficking [63].

## 5. Conclusions

The development of various types of fluorescent materials for targeting mitochondria has contributed to elucidating mitochondrial biology. The structural insights and systematic studies for targeting mitochondria have revealed that regulation of hydrophobicity and charge are critical in mitochondrial targeting efficiency in chemical and peptide-based approaches. The application of mitochondrial targeting probes such as chemicals or nanomaterials has provided a therapeutic potential as a theragnostic tool for cancer treatment. Versatile mitochondrial probes allow real-time visualization of mitochondria and related biological processes in in vitro and in vivo systems, which suggests they are useful tools for mitochondrial biology in health and disease.

## Figures and Tables

**Table 1:** Summary of the chemical probes for targeting various types of mitochondrial metabolites.

Structure ^1^	Metabolite	Ex/Em ^2^
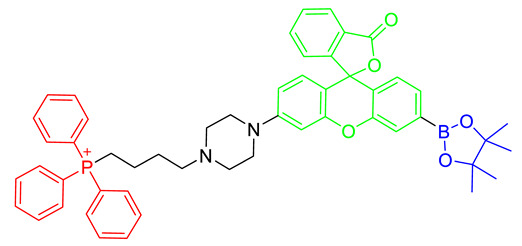 MitoPY1 [36,37]	Mitochondrial H_2_O_2_	503 nm/ 510–750 nm
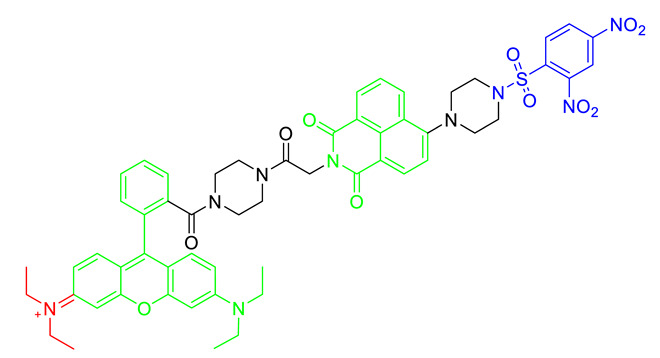 MT-1 [38]	Mitochondrial thiol, Mitochondrial glutathione	395 nm (one-photon) or 800 nm (two-photon)/ 589 nm
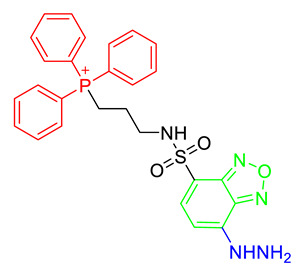 Mito-FMP [39]	Malondialdehyde	373 nm/554 nm
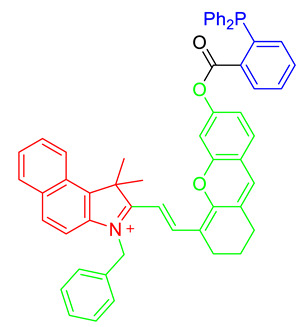 MitoHNO [40]	HNO	690 nm/727 nm

^1^ In the chemical structures, green, red, and blue indicate the fluorophore, the mitochondrial targeting lipophilic cationic moiety, and the recognition site for individual metabolite, respectively. ^2^ Ex means excitation wavelength and Em means emission wavelength of fluorophores for metabolite detection.

## Data Availability

No new data were created or analyzed in this study. Data sharing is not applicable to this article.

## References

[1] Jonckheere A.I., Smeitink J.A., Rodenburg R.J. (2012). Mitochondrial ATP synthase: Architecture, function and pathology. J. Inherit. Metab. Dis..

[2] Tan J.X., Finkel T. (2020). Mitochondria as intracellular signaling platforms in health and disease. J. Cell Biol..

[3] Handy D.E., Loscalzo J. (2012). Redox regulation of mitochondrial function. Antioxid. Redox Signal..

[4] Wisnovsky S., Lei E.K., Jean S.R., Kelley S.O. (2016). Mitochondrial Chemical Biology: New Probes Elucidate the Secrets of the Powerhouse of the Cell. Cell Chem. Biol..

[5] Vamecq J., Dessein A.F., Fontaine M., Briand G., Porchet N., Latruffe N., Andreolotti P., Cherkaoui-Malki M. (2012). Mitochondrial dysfunction and lipid homeostasis. Curr. Drug Metab..

[6] Stehling O., Lill R. (2013). The role of mitochondria in cellular iron-sulfur protein biogenesis: Mechanisms, connected processes, and diseases. Cold Spring Harb. Perspect. Biol..

[7] Burke P.J. (2017). Mitochondria, Bioenergetics and Apoptosis in Cancer. Trends Cancer.

[8] Duchen M.R. (2004). Mitochondria in health and disease: Perspectives on a new mitochondrial biology. Mol. Asp. Med..

[9] Norat P., Soldozy S., Sokolowski J.D., Gorick C.M., Kumar J.S., Chae Y., Yagmurlu K., Prada F., Walker M., Levitt M.R. (2020). Mitochondrial dysfunction in neurological disorders: Exploring mitochondrial transplantation. NPJ Regen. Med..

[10] Frey T.G., Mannella C.A. (2000). The internal structure of mitochondria. Trends Biochem. Sci..

[11] Chen H., Chan D.C. (2009). Mitochondrial dynamics—Fusion, fission, movement, and mitophagy—In neurodegenerative diseases. Hum. Mol. Genet..

[12] Westermann B. (2010). Mitochondrial fusion and fission in cell life and death. Nat. Rev. Mol. Cell Biol..

[13] Li X., Zhao Y., Yin J., Lin W. (2020). Organic fluorescent probes for detecting mitochondrial membrane potential. Coord. Chem. Rev..

[14] Hoffman R.M. (2005). Advantages of multi-color fluorescent proteins for whole-body and in vivo cellular imaging. J. Biomed. Opt..

[15] Wolfbeis O.S. (2015). An overview of nanoparticles commonly used in fluorescent bioimaging. Chem. Soc. Rev..

[16] Mitra K., Lippincott-Schwartz J. (2010). Analysis of mitochondrial dynamics and functions using imaging approaches. Curr. Protoc. Cell Biol..

[17] Sun N., Malide D., Liu J., Rovira I.I., Combs C.A., Finkel T. (2017). A fluorescence-based imaging method to measure in vitro and in vivo mitophagy using mt-Keima. Nat. Protoc..

[18] Jakobs S. (2006). High resolution imaging of live mitochondria. Biochim. Biophys. Acta.

[19] Samanta S., He Y., Sharma A., Kim J., Pan W., Yang Z., Li J., Yan W., Liu L., Qu J. (2019). Fluorescent Probes for Nanoscopic Imaging of Mitochondria. Chem.

[20] Murphy M.P. (2008). Targeting lipophilic cations to mitochondria. Biochim. Biophys. Acta.

[21] Lincoln R., Greene L.E., Zhang W., Louisia S., Cosa G. (2017). Mitochondria Alkylation and Cellular Trafficking Mapped with a Lipophilic BODIPY-Acrolein Fluorogenic Probe. J. Am. Chem Soc..

[22] Petrat F., Pindiur S., Kirsch M., de Groot H. (2003). “Mitochondrial” photochemical drugs do not release toxic amounts of 1O(2) within the mitochondrial matrix space. Arch. Biochem. Biophys..

[23] Li H., Xin C., Zhang G., Han X., Qin W., Zhang C.-W., Yu C., Jing S., Li L., Huang W. (2019). A mitochondria-targeted two-photon fluorogenic probe for the dual-imaging of viscosity and H2O2 levels in Parkinson’s disease models. J. Mater. Chem. B.

[24] Saha P.C., Chatterjee T., Pattanayak R., Das R.S., Mukherjee A., Bhattacharyya M., Guha S. (2019). Targeting and Imaging of Mitochondria Using Near-Infrared Cyanine Dye and Its Application to Multicolor Imaging. ACS Omega.

[25] Levi S., Corsi B., Bosisio M., Invernizzi R., Volz A., Sanford D., Arosio P., Drysdale J. (2001). A human mitochondrial ferritin encoded by an intronless gene. J. Biol. Chem..

[26] Samudio I., Konopleva M., Hail N., Shi Y.X., McQueen T., Hsu T., Evans R., Honda T., Gribble G.W., Sporn M. (2005). 2-Cyano-3,12-dioxooleana-1,9-dien-28-imidazolide (CDDO-Im) directly targets mitochondrial glutathione to induce apoptosis in pancreatic cancer. J. Biol. Chem..

[27] Zhou R., Yazdi A.S., Menu P., Tschopp J. (2011). A role for mitochondria in NLRP3 inflammasome activation. Nature.

[28] Bates M., Jones S.A., Zhuang X. (2013). Preparation of photoswitchable labeled antibodies for STORM imaging. Cold Spring Harb. Protoc..

[29] Choi S.K., Rho J., Yoon S.E., Seok J.H., Kim H., Min J., Yoon W., Lee S., Yun H., Kwon O.P. (2020). Full Color Tunable Aggregation-Induced Emission Luminogen for Bioimaging Based on an Indolizine Molecular Framework. Bioconjug Chem..

[30] Choi S.-K., Lee Y., Yoon S.E., Choi H., Kim J., Kim J.H., Lee S., Kim W., Kim E. (2021). A tetrazine-fused aggregation induced emission luminogen for bioorthogonal fluorogenic bioprobe. Sens. Actuators B Chem..

[31] Sung J., Rho J.G., Jeon G.G., Chu Y., Min J.S., Lee S., Kim J.H., Kim W., Kim E. (2019). A New Infrared Probe Targeting Mitochondria via Regulation of Molecular Hydrophobicity. Bioconjug Chem..

[32] Chu Y., Shin M.C., Sung J., Park J., Kim E., Lee S. (2019). Development of Theragnostic Tool Using NIR Fluorescence Probe Targeting Mitochondria in Glioma Cells. Bioconjug Chem..

[33] Xiao H., Li P., Zhang W., Tang B. (2016). An ultrasensitive near-infrared ratiometric fluorescent probe for imaging mitochondrial polarity in live cells and in vivo. Chem. Sci..

[34] Ma C., Xia F., Kelley S.O. (2020). Mitochondrial Targeting of Probes and Therapeutics to the Powerhouse of the Cell. Bioconjug Chem..

[35] Zielonka J., Joseph J., Sikora A., Hardy M., Ouari O., Vasquez-Vivar J., Cheng G., Lopez M., Kalyanaraman B. (2017). Mitochondria-Targeted Triphenylphosphonium-Based Compounds: Syntheses, Mechanisms of Action, and Therapeutic and Diagnostic Applications. Chem. Rev..

[36] Dickinson B.C., Chang C.J. (2008). A targetable fluorescent probe for imaging hydrogen peroxide in the mitochondria of living cells. J. Am. Chem. Soc..

[37] Dickinson B.C., Lin V.S., Chang C.J. (2013). Preparation and use of MitoPY1 for imaging hydrogen peroxide in mitochondria of live cells. Nat. Protoc..

[38] Liu Z., Wang Q., Wang H., Su W., Dong S. (2020). A FRET Based Two-Photon Fluorescent Probe for Visualizing Mitochondrial Thiols of Living Cells and Tissues. Sensors.

[39] He L., Yang X., Xu K., Lin W. (2017). A mitochondria-targeted fluorescent probe for imaging endogenous malondialdehyde in HeLa cells and onion tissues. Chem. Commun..

[40] Gong X., Yang X.-F., Zhong Y., Chen Y., Li Z. (2016). A mitochondria-targetable near-infrared fluorescent probe for imaging nitroxyl (HNO) in living cells. Dyes Pigment..

[41] Perry S.W., Norman J.P., Barbieri J., Brown E.B., Gelbard H.A. (2011). Mitochondrial membrane potential probes and the proton gradient: A practical usage guide. Biotechniques.

[42] Lin M.T., Beal M.F. (2006). Mitochondrial dysfunction and oxidative stress in neurodegenerative diseases. Nature.

[43] Keeney P.M., Xie J., Capaldi R.A., Bennett J.P. (2006). Parkinson’s disease brain mitochondrial complex I has oxidatively damaged subunits and is functionally impaired and misassembled. J. Neurosci..

[44] Sivandzade F., Bhalerao A., Cucullo L. (2019). Analysis of the Mitochondrial Membrane Potential Using the Cationic JC-1 Dye as a Sensitive Fluorescent Probe. Bio Protoc..

[45] Zhao N., Chen S., Hong Y., Tang B.Z. (2015). A red emitting mitochondria-targeted AIE probe as an indicator for membrane potential and mouse sperm activity. Chem. Commun..

[46] Horton K.L., Stewart K.M., Fonseca S.B., Guo Q., Kelley S.O. (2008). Mitochondria-penetrating peptides. Chem. Biol..

[47] Kang Y.C., Son M., Kang S., Im S., Piao Y., Lim K.S., Song M.Y., Park K.S., Kim Y.H., Pak Y.K. (2018). Cell-penetrating artificial mitochondria-targeting peptide-conjugated metallothionein 1A alleviates mitochondrial damage in Parkinson’s disease models. Exp. Mol. Med..

[48] Fonseca S.B., Pereira M.P., Mourtada R., Gronda M., Horton K.L., Hurren R., Minden M.D., Schimmer A.D., Kelley S.O. (2011). Rerouting chlorambucil to mitochondria combats drug deactivation and resistance in cancer cells. Chem. Biol..

[49] Abad M.F., Di Benedetto G., Magalhaes P.J., Filippin L., Pozzan T. (2004). Mitochondrial pH monitored by a new engineered green fluorescent protein mutant. J. Biol. Chem..

[50] Youle R.J., Narendra D.P. (2011). Mechanisms of mitophagy. Nat. Rev. Mol. Cell Biol..

[51] Kogure T., Karasawa S., Araki T., Saito K., Kinjo M., Miyawaki A. (2006). A fluorescent variant of a protein from the stony coral Montipora facilitates dual-color single-laser fluorescence cross-correlation spectroscopy. Nat. Biotechnol..

[52] Bingol B., Tea J.S., Phu L., Reichelt M., Bakalarski C.E., Song Q., Foreman O., Kirkpatrick D.S., Sheng M. (2014). The mitochondrial deubiquitinase USP30 opposes parkin-mediated mitophagy. Nature.

[53] Sun N., Yun J., Liu J., Malide D., Liu C., Rovira I.I., Holmstrom K.M., Fergusson M.M., Yoo Y.H., Combs C.A. (2015). Measuring In Vivo Mitophagy. Mol. Cell.

[54] Prescher J.A., Bertozzi C.R. (2005). Chemistry in living systems. Nat. Chem. Biol..

[55] Oliveira B.L., Guo Z., Bernardes G.J.L. (2017). Inverse electron demand Diels-Alder reactions in chemical biology. Chem. Soc. Rev..

[56] Kim E., Koo H. (2019). Biomedical applications of copper-free click chemistry: In vitro, in vivo, and ex vivo. Chem. Sci..

[57] Saal K.A., Richter F., Rehling P., Rizzoli S.O. (2018). Combined Use of Unnatural Amino Acids Enables Dual-Color Super-Resolution Imaging of Proteins via Click Chemistry. ACS Nano.

[58] van Loo G., Saelens X., van Gurp M., MacFarlane M., Martin S.J., Vandenabeele P. (2002). The role of mitochondrial factors in apoptosis: A Russian roulette with more than one bullet. Cell Death Differ..

[59] Kwon H.J., Cha M.Y., Kim D., Kim D.K., Soh M., Shin K., Hyeon T., Mook-Jung I. (2016). Mitochondria-Targeting Ceria Nanoparticles as Antioxidants for Alzheimer’s Disease. ACS Nano.

[60] Liu Z., Pei H., Zhang L., Tian Y. (2018). Mitochondria-Targeted DNA Nanoprobe for Real-Time Imaging and Simultaneous Quantification of Ca(2+) and pH in Neurons. ACS Nano.

[61] Feng Z., Ma Y., Li B., He L., Wang Q., Huang J., Liu J., Yang X., Wang K. (2019). Mitochondria targeted self-assembled ratiometric fluorescent nanoprobes for pH imaging in living cells. Anal. Methods.

[62] Kim K.Y., Jin H., Park J., Jung S.H., Lee J.H., Park H., Kim S.K., Bae J., Jung J.H. (2017). Mitochondria-targeting self-assembled nanoparticles derived from triphenylphosphonium-conjugated cyanostilbene enable site-specific imaging and anticancer drug delivery. Nano Res..

[63] Chakraborty A., Jana N.R. (2015). Design and Synthesis of Triphenylphosphonium Functionalized Nanoparticle Probe for Mitochondria Targeting and Imaging. J. Phys. Chem. C.

[64] Wang J.-L., Zhang L., Zhao M.-J., Zhang T., Liu Y., Jiang F.-L. (2021). Mitochondria-Targeted BODIPY Nanoparticles for Enhanced Photothermal and Photoacoustic Imaging In Vivo. ACS Appl. Bio Mater..

[65] Zeng W.N., Yu Q.P., Wang D., Liu J.L., Yang Q.J., Zhou Z.K., Zeng Y.P. (2021). Mitochondria-targeting graphene oxide nanocomposites for fluorescence imaging-guided synergistic phototherapy of drug-resistant osteosarcoma. J. Nanobiotechnol..

